# Event and Comment

**Published:** 1923-04

**Authors:** 


### DENTAL REGISTER

THE MAGAZINE OF DENTISTRY

Vol. LXXVII APRIL, 1923 No. 4

### EVENT AND COMMENT

BY VOTRE AMI DENTAIRE

---

The spring time is when the dental practice in the country takes on new life. Many people in the country begin to come into the villages for shopping and to attend court on a court day, to pay their taxes, etc., and to see their dentist too. Court days are an old institution in Kentucky and make a busy day for the townspeople. It is not a good day for a dental man to visit the town for his customer is too vitally busy and engaged to spend the time otherwise than operating and attending to patients. But if you can not avoid being in a town on court day it is interesting anyway. To see the autos and buggies parked about the public square shows one the immense importance of the town to the surrounding farm lands. The mud on the wheels tells much and suggests tall timber. Court day at the dental office is important in its way, for appointments are made for further work which may take the rest of the summer to finish.

Later on in the early summer, the town having a court day makes a romantic setting for social affairs of the folk of the locality.

---

The new building which is going on in the various cities and towns of the United States should eventually make a good market of new dental equipment. Office buildings are being provided now with electricity, water, compressed air and gas at convenient points on the floor or baseboard to accommodate new equipment as it is made today. The Unit Pedestal for instance takes care of all the motive power, light, water, drain and gas heat used at the chair. Architects constructing new office buildings are informed on these requirements and are incorporating conveniences for professional equipment that were lacking in the older buildings.

---

### MESSAGE OF THE GOOD FAIRY

I spring from the spray on the tip of the crest
  Of the billows from out of the blue.
I am bringing a message that says you are blest
  By the love that your friends bear for you.
If my arms were as long as the wings of the wind
  And I'd stretch them wide away,
I never could hold half the blessings I'm told
  Your friends are wishing to day.
As long as I stand with this smile on my face
  And my arms outstretched so high,
If you'll think of the friend that sent me to you,
  You can never be blue if you try.

—*Leo S. Robinson.*

The above verse was written by Leo S. Robinson, the maker of Robinson bristle discs, at Alameda, California. The discs are good and so is the verse. Mr. Robinson in his reflective mood is a writer of verse for greeting cards.

---

The demonstration of Jelenko Golds given by Mr. C. H. Ackerman at the Hotel Sinton, Cincinnati, on April 3d and 4th, was enthusiastically received. Mr. Ackerman demonstrated the best methods of procedure in gold casting clasps—plates, inlays, etc. His time was completely taken up and a number of dentists who came late only saw part of the display, for Mr. Ackerman had to leave at the end of his second day here for Louisville. Mr. Ackerman is a very interesting demonstrator and is well versed in his work and is besides very interesting personally, as he has studied much and traveled a great deal.

Jelenko Golds are a very valuable adjunct to dentistry and the profession demonstrated their appreciation of this value by turning out to see the wonderful demonstration put on by Mr. Ackerman and by increasing use of the Jelenko materials.

